# Survival of paediatric dialysis patients in Türkiye over a three-year follow-up: findings from national database

**DOI:** 10.1007/s00467-026-07264-z

**Published:** 2026-04-08

**Authors:** Gülşah Kaya Aksoy, Sema Akman, Fatih Kacıroğlu, Bağdagül Aksu, İpek Kaplan Bulut, Gökçen Erfidan, Eda Karadoğan, Hülya Nalçacıoğlu, Ayşe Seda Pınarbaşı, Şenay Zırhlı Selçuk, Sebahat Tülpar, Sibel Yel, Betül Pehlivan Zorlu, Harika Alpay, Ayşe Balat, Esra Baskın, Aysun Karabay Bayazıt, Umut Selda Bayrakçı, Mehmet Bülbül, Cengiz Candan, Nur Canpolat, Evrim Kargın Çakıcı, Elif Çomak, Ali Delibaş, Belde Kasap Demir, Ali Düzova, Osman Dönmez, Hakan Erdoğan, Pelin Ertan, Sevcan Azime Bakkaloğlu, Mustafa Koyun, Z. Aytül Noyan, Zeynep Birsin Özçakar, Emine Neşe Özkayın, Sevgi Yavuz, Dilek Yılmaz, Selçuk Yüksel

**Affiliations:** 1 Faculty of Medicine, Department of Pediatric Nephrology, Akdeniz University, Antalya, Türkiye; 2 Head of Tissue, General Directorate of Health Services, Organ Transplantation and Dialysis Services, Ankara, Türkiye; 3 İstanbul Faculty of Medicine, Department of Pediatric Nephrology, İstanbul University, Istanbul, Türkiye; 4 Faculty of Medicine, Department of Pediatric Nephrology, Ege University, Izmir, Türkiye; 5 Diyarbakır Gazi Yaşargil Hospital, Pediatric Nephrology Unit, Diyarbakır, Türkiye; 6 Epidemiologist Republic of Türkiye Ministry of Health, Antalya Provincial Health Administration, Antalya, Türkiye; 7 Faculty of Medicine, Department of Pediatric Nephrology, Ondokuz Mayıs University, Samsun, Türkiye; 8 Diyarbakır Childrens Hospital, Pediatric Nephrology Unit, Diyarbakır, Türkiye; 9 Faculty of Medicine, Department of Pediatric Nephrology, İnönü University, Malatya, Türkiye; 10 Bakırköy Dr. Sadi Konuk Research and Training Hospital, Department of Pediatric Nephrology, Istanbul, Türkiye; 11 Faculty of Medicine, Department of Pediatric Nephrology, Erciyes University, Kayseri, Türkiye; 12 Faculty of Medicine, Department of Pediatric Nephrology, Uz Hospital, İzmir Dr Behçet, İzmir, Türkiye; 13 Department of Pediatric Nephrology, Marmara University Faculty of Medicine, Istanbul, Türkiye; 14 Faculty of Medicine, Division of Pediatric Nephrology, Gaziantep University, Gaziantep, Türkiye; 15 Faculty of Medicine, Department of Pediatric Nephrology, Baskent University, Ankara, Türkiye; 16 Faculty of Medicine, Department of Pediatric Nephrology, Çukurova University, Adana, Türkiye; 17 Bilkent City Hospital, Pediatric Nephrology Unit, Ankara, Türkiye; 18 Pediatric Nephrology Department, Ankara Etlik City Hospital, Ankara, Türkiye; 19 Faculty of Medicine, Department of Pediatric Nephrology, İstanbul Medeniyet University, Istanbul, Türkiye; 20 Department of Pediatric Nephrology, İstanbul University-Cerrahpaşa, Cerrahpasa Faculty of Medicine, Istanbul, Türkiye; 21 Faculty of Medicine, Department of Pediatric Nephrology, Mersin University, Mersin, Türkiye; 22 Faculty of Medicine, Department of Pediatric Nephrology, İzmir Katip Çelebi University, İzmir, Türkiye; 23 Division of Pediatric Nephrology, Hacettepe University Faculty of Medicine, Ankara, Türkiye; 24 Faculty of Medicine, Department of Pediatric Nephrology, Uludağ University, Bursa, Türkiye; 25 Department of Pediatric Nephrology, Bursa City Hospital, Bursa, Türkiye; 26 Faculty of Medicine, Department of Pediatric Nephrology, Manisa Celal Bayar University, Manisa, Türkiye; 27 Faculty of Medicine, Department of Pediatric Nephrology, Gazi University, Ankara, Türkiye; 28 Department of Pediatric Nephrology, Başkent University, Adana Dr Turgut Noyan Training and Research Center, Adana, Türkiye; 29 Division of Pediatric Nephrology, Ankara University School of Medicine, Ankara, Türkiye; 30 Faculty of Medicine, Department of Pediatric Nephrology, Trakya University, Edirne, Türkiye; 31 Başakşehir Çam Ve Sakura City Hospital, Pediatric Nephrology Unit, Istanbul, Türkiye; 32 Faculty of Medicine, Department of Pediatric Nephrology, Aydın Adnan Menderes University, Aydın, Türkiye; 33 Department of Pediatric Nephrology, Çanakkale Onsekiz Mart University Faculty of Medicine, Denizli, Türkiye

**Keywords:** Children, Kidney failure, Stage 5 chronic kidney disease, Peritoneal dialysis, Hemodialysis, Survival outcomes

## Abstract

**Background:**

Dialysis is a life-saving treatment for children with stage 5 chronic kidney disease (CKD), but it is associated with a high long-term mortality that varies between countries. The aim of this study was to compare 3-year survival rates of children and adolescents on peritoneal dialysis (PD) and hemodialysis (HD) based on national data from Türkiye.

**Methods:**

We analyzed the national registry data to assess survival rates among pediatric patients on dialysis in Türkiye. Kaplan–Meier and Cox proportional hazards models compared PD and HD survival outcomes, adjusting for age, period of initial dialysis commencement, and primary diagnosis.

**Results:**

The study included 1,002 children with a mean age of 12.71 ± 4.80 years. The overall mortality rate among pediatric dialysis patients was 25.8 deaths per 1000 patient-years, with adjusted analyses showing a 5-year survival advantage for PD over HD (adjusted *p* = 0.000001). The median adjusted survival probability was 0.89 (IQR 0.26) for PD and 0.83 (IQR 0.08) for HD, and PD demonstrated superior outcomes particularly in non-CAKUT children aged 6–12 and > 12 years (*p* = 0.004 and 0.013, respectively). Multivariate Cox regression analysis revealed that haemodialysis, initiation of dialysis between 2006 and 2018, and younger age (< 12 years) were independently associated with an increased risk of mortality in paediatric dialysis patients.

**Conclusion:**

In Türkiye, peritoneal dialysis has been shown to be associated with better survival rates than haemodialysis in children with stage 5 CKD, particularly among younger patients.

**Graphical Abstract:**

**Figure:**
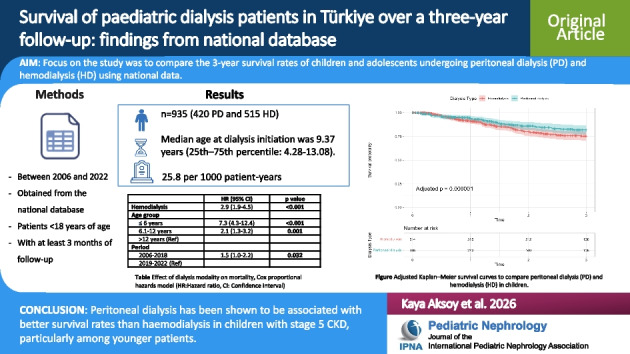
A higher resolution version of Graphical abstract is available as Supplementary information

**Supplementary Information:**

The online version contains supplementary material available at 10.1007/s00467-026-07264-z.

## Introductıon

In pediatric patients with stage 5 chronic kidney disease (CKD), dialysis provides fluid management, maintains electrolyte balance, and removes nitrogenous waste products. Despite these benefits and the advance of technology, the mortality rate in pediatric patients on dialysis is 30 times higher compared to the healthy population [1]. The most frequent causes of death are from cardiovascular complications and infections [2, 3]. A nationwide cohort study from Japan has reported in 2022, a 1-year and a 5-year survival rate of 97.2% and 92.5%, respectively [4]. Data from the Australia and New Zealand Dialysis and Transplant Registry states that children aged ≤ 5 years treated by kidney replacement therapy have survival rates of 93% after one year, 86% after five years, and 83% after ten years [4].

Hemodialysis (HD) has some disadvantages: the complications related to vascular access and increased exposure to the hospital environment. On the other hand, peritoneal dialysis (PD) has more infection-related complications. Several European and American registry studies have reported that the risk of death among pediatric patients on PD is significantly lower than in those treated with HD [5–7]. Again, it has to be taken into consideration in the U.S. cohort that by far the greatest advantage of PD was in children aged less than 5 years [5]. Most studies indicate that the mortality rate is higher at an early age, and it does not depend on the dialysis method being followed [4, 5, 8]. The McDonald et al. study showed the risk of death was four times greater in children less than 1 year when compared with more than 15 years [8].

Projections regarding survival of paediatric patients with dialysis vary quite significantly from one country to another. Mortality rates vary between 15.8 and 68.6 deaths per 1,000 patient-years, showing wide variation in outcomes worldwide [2–14]. A study of PD patients performed in our country in 2005 found an overall mortality rate of 83 deaths per 1.000 patient-years [15]. This study was conducted to present comprehensive, nationally representative data concerning pediatric patients undergoing chronic dialysis in Türkiye.

## Methods

Data on pediatric patients who received dialysis at any time between October 2006 and October 2022 were obtained through a retrospective analysis of the National Database under the Transplantation, Dialysis, and Monitoring Systems infrastructure of the Ministry of Health. This included all patients younger than 18 years old at initiation of dialysis with a regular follow-up period of at least 3 months. In our analysis, we assumed that the missing data were Missing Completely at Random (MCAR) and therefore excluded cases with missing values. It is posited that, in a national registry-based real-world dataset, missingness is unlikely to be completely at random. However, due to the paucity of information regarding the mechanisms of missing data and the absence of auxiliary variables required for more advanced imputation methods, this approach was considered the most feasible option.The 3-year follow-up included approximately 25% of the original cohort, mainly because follow-up data were not recorded for a substantial proportion of patients or because some patients had not yet reached the 3-year follow-up period. In this retrospective analysis, only patients who were continuously treated with dialysis throughout the follow-up period were included; patients who underwent kidney transplantation (including those whose grafts were lost and who returned to dialysis) during the follow-up period were excluded. Ethical approval was taken from the Clinical Research Ethics Committee of the Akdeniz University Faculty of Medicine (16.03.2022–136).

Dialysis onset was defined as the initiation of any form of chronic dialysis therapy, and total dialysis duration was calculated from this onset without stratification by dialysis modality. Patients were classified as HD or PD according to the dialysis modality they were receiving at the time of study inclusion. The following data were collected: sex, age at initiation of dialysis, underlying primary kidney disease, serum levels of hemoglobin, albumin, C-reactive protein (CRP), and parathormon (PTH), treatment requirements including erythropoiesis-stimulating agents (ESA). Data was recorded annually during the follow-up period.

### Statistical analysis

Descriptive statistics are presented as frequency and percentage for categorical variables and as mean ± SD for normally distributed continuous variables or median (25th–75th percentiles) for non-normally distributed continuous variables. The normality of distribution was checked by the Shapiro–Wilk test, histogram, and Q–Q graphics. Group comparisons were performed for both categorical and continuous variables in the HD and PD groups. The Chi-squared test or Fisher's exact test was used to analyze relationships between categorical variables. For the continuous variables with a normal distribution, the Student's t-test was applied, while the Mann–Whitney U test was applied for those with non-normal distribution. A p value of < 0.05 was considered to be statistically significant. All-cause mortality rates were estimated by dividing the total number of deaths by the total person-time at risk, and are reported as events per 1,000 patient-years. Kaplan–Meier survival estimates were calculated, and factors potentially influencing survival were evaluated using Cox proportional hazards regression analysis. Both multivariate analysis and stratified analyses were used to control for confounding factors, after investigating the presence of effect modifiers using the Breslow–Day test and the interaction term added to the multivariate analysis, where none were detected. Statistical analysis was performed using SPSS version 21.0 package program for Windows (IBM, Armonk, NY). The Kaplan–Meier graphs were created using R version 4.5.0.

## Results

### Study population

The study population consisted of 1,002 children: 510 (50.9%) boys, with a mean age of 12.71 ± 4.80 years. The median age at the time of dialysis initiation was 9.40 (25th–75th percentiles: 4.13–13.14) years and median follow-up time 1.56 (25th–75th percentiles: 0.88–3.14) years. Information on the primary kidney disease diagnosis could not be obtained in 47% of the patients under study (*n* = 453). Accordingly, the distribution of diagnoses was analyzed on the remaining 549 patients (Table 1). The distribution of diagnoses between HD and PD was found to be similar in the study groups (*p* = 0.156) (Table 1). At the time of the survey, there were 539 patients (53.8%) on HD and 463 patients (46.2%) on PD, yielding a HD-to-PD ratio of 1.16. Among the patients on HD, 91 (16.8%) previously had been treated with PD with a median duration of 2.9 years (interquartile range (IQR): 3.33). Conversely, among the patients on PD, 14 (3.0%) had been previously treated with HD with a median duration of 4.69 months (IQR: 23.97).

**Table 1:** Demographic and clinical characteristics of the study population by dialysis modality

Variables	Peritoneal dialysis (*n* = 463)	Hemodialysis (*n* = 539)	*p*
Mean age at time of study (years)	10.06 ± 4.85	14.62 ± 3.84	< 0.001
Sex, boys (%)	235 (51.4)	265 (50.5)	0.508
Median follow-up time (years)	1.87 (0.97–3.50)	1.42 (0.59–3.06)	< 0.001
Median age at onset of dialysis (years)	5.94 (1.41–10.41)	11.46 (7.86–14.54)	< 0.001
Age categories at the initiation of dialysis, (%)			
< 6 years 6–12 years > 12 years	124 (27.1) 120 (26.3) 213 (46.6)	26 (5.0) 91 (17.3) 408 (77.7)	< 0.001
Primary kidney disease diagnosis, n (%)			
CAKUT Glomerular diseases Tubulointerstitial diseases Cystic kidney diseases Alport syndrome and other hereditary diseases Oxalosis and urolithiasis Other Unknown	116 (25.0) 42 (9.1) 10 (2.2) 27 (5.8) 14 (3.0) 9 (1.9) 88 (19.0) 157 (34.1)	63 (11.7) 32 (5.9) 6 (1.1) 5 (0.9) 12 (2.2) 6 (1.1) 89 (16.5) 326 (60.6)	0.156

*CAKUT* Congenital anomalies of the kidney and urinary tract

### Features of hemodialysis and peritoneal dialysis

At the initiation of dialysis, the distribution of vascular access types in HD patients was as follows: 114 patients (21.2%) used a temporary non-tunneled catheter, 324 (60.1%) had a permanent tunneled catheter, 80 (14.8%) had an AV fistula, and 3 (0.6%) had AV grafts.

Among the patients, 504 (93.5%) received standard HD, 10 (1.9%) hemodiafiltration, and 6 (1.1%) home HD. In 76.8% of the patients, dialysis was being performed three times a week, whereas in 6.3% it was more than three times a week. At PD initiation, 39.1% of patients were initiated on continuous ambulatory peritoneal dialysis (CAPD) and 60.9% on automated peritoneal dialysis (APD).

### Comparison of patients on hemodialysis and peritoneal dialysis

The sex distribution was not significantly different between the PD and HD groups (*p* = 0.508). However, the age at initiation of dialysis was significantly lower in the PD group compared with the HD group (5.94 years vs. 11.46 years; *p* < 0.001). In addition, the percentage of patients below 6 years of age and those in the age group 6–12 years was significantly higher in the PD group, whereas the percentage of patients above 12 years of age was significantly higher in the HD group (*p* < 0.001) (Table 1). Notably, among patients ≤ 6 years of age in the 2006–2018 period, only one patient underwent HD.

### Patient survival

**Fig. 1:**
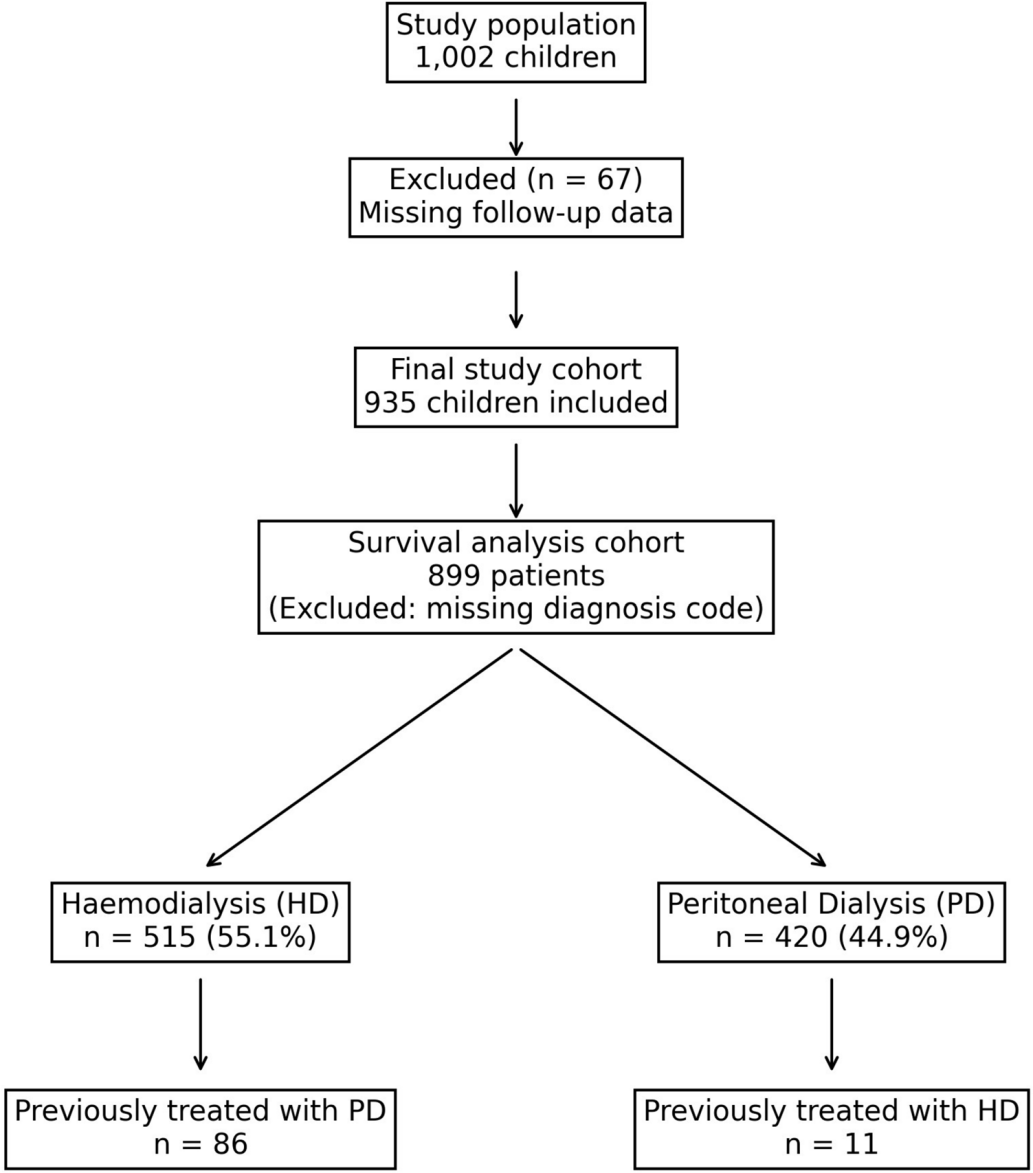
Study population flowchart

From 2006 through 2022, the mortality rate among pediatric patients undergoing dialysis treatment was 25.8 per 1000 patient-years. An adjusted Kaplan–Meier curve, adjusted for underlying diagnosis (CAKUT vs. non-CAKUT), year of starting dialysis and age, was plotted based on the Cox regression model and stratified by dialysis modality. The adjusted median survival probability at 3 years was 0.83 (IQR: 0.08) for HD patients and 0.89 (IQR: 0.26) for PD patients. While the median survival probabilities were similar, the overall adjusted survival distributions demonstrated significantly better survival at 3 years in PD patients compared to HD (adjusted *p* = 0.000001) (Fig. 1).

**Fig. 2:**
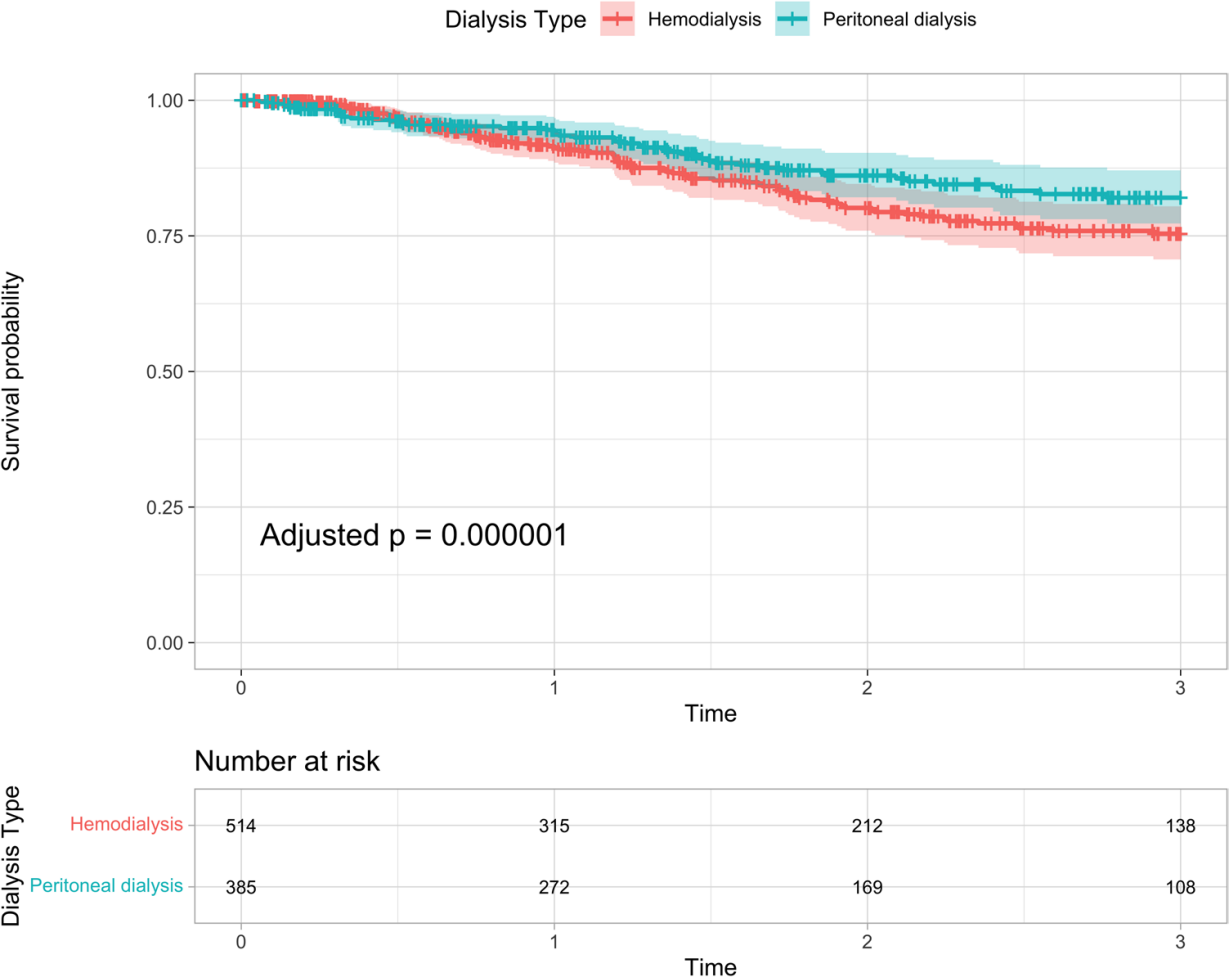
Adjusted Kaplan–Meier survival curves comparing peritoneal dialysis (PD) and hemodialysis (HD) in children. The p value was derived from multivariable Cox proportional hazards model adjusted for age category, study period, and presence of CAKUT

Survival results were also analyzed by stratification of the patients according to underlying diagnosis (CAKUT vs. non-CAKUT) and age category. In children with CAKUT, PD was associated with better survival than HD in those aged ≤ 6 years (*p* = 0.034); however, the survival advantage was no longer observed in the 6.1–12 year age group (*p* = 0.678). Notably, no deaths were observed in the PD group among patients aged > 12 years during the follow-up period. In the non-CAKUT group, PD was associated with significantly better survival in the age categories 6–12 years and > 12 years (*p* = 0.004 and 0.013, respectively), while there was no significant difference found in ≤ 6 years (*p* = 0.275) (Fig. 2).

**Fig. 3:**
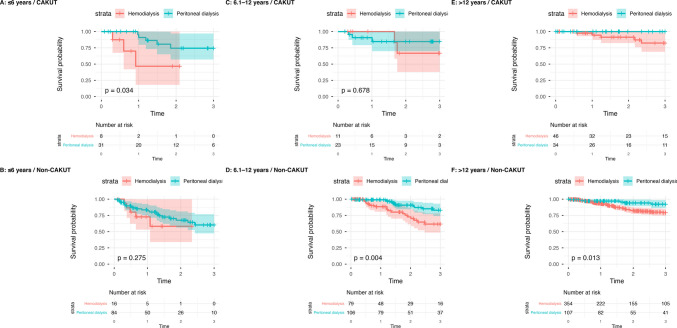
Kaplan–Meier survival curves comparing peritoneal dialysis (PD) and hemodialysis (HD) in pediatric patients, stratified by age group and primary diagnosis (CAKUT vs. non-CAKUT). Panels A–C represent patients with CAKUT: (**A**) ≤ 6 years, (**C**) 6.1–12 years, and (**E**) > 12 years. Panels B, D, and F represent patients with non-CAKUT diagnoses: (**B**) ≤ 6 years, (**D**) 6.1–12 years, and (**F**) > 12 years. p values were obtained using the log rank test

**Fig. 4:**
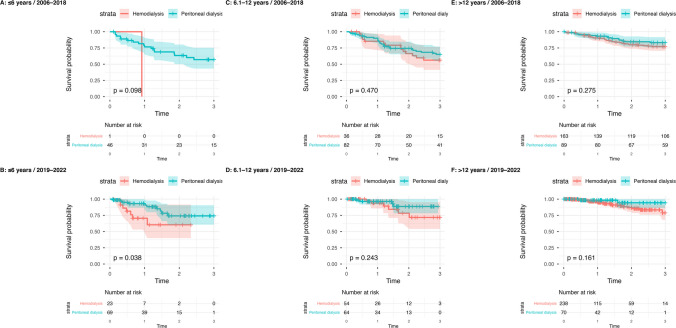
Kaplan–Meier survival plots of children on dialysis, comparing peritoneal dialysis (PD) vs. hemodialysis (HD) in three age groups in two eras. Each panel (A–F) is for a single age group in an era, with PD (blue curves) and HD (red curves) plotted for that age subgroup. (**A**,** B**) Age ≤ 6 years in 2006–2018 (A) and 2019–2022 (B). (**C**,** D**) Children between 6.1–12 years in 2006–2018 (C) and 2019–2022 (D). (**E**,** F**) Children over 12 years in 2006–2018 (E) and 2019–2022 (F). p values were obtained using the log rank test

Among patients ≤ 6 years, survival comparison between PD and HD was performed only for the 2019–2022 period, during which PD was associated with significantly better survival than HD (*p* = 0.038). In 6–12 years of children, PD and HD survival outcomes were comparable in both time periods, and there were no statistically significant differences in 2006–2018 (*p* = 0.470) or 2019–2022 (*p* = 0.243). Similarly, in patients > 12 years, the difference in survival between PD and HD was not statistically significant in either period (2006–2018: *p* = 0.275; 2019–2022: *p* = 0.161) (Figs. 3 and 4).

Multivariate Cox proportional hazards regression analysis identified several independent predictors of survival in children undergoing dialysis. The multivariate Cox proportional hazards model demonstrated that hemodialysis [HR (%95 CI) = 2.9 (1.9–4.5), *p* < 0.001] and dialysis initiation between 2006 and 2018 [HR (%95 CI) = 1.5 (1.0–2.2), *p* = 0.032] were significantly associated with mortality. Furthermore, younger age groups (≤ 6 years and 6–12 years) were also found to be significantly associated with an increased risk of mortality [HR (%95 CI) respectively = 7.3 (4.3–12.4), 2.1 (1.3–3.2), *p* value respectively < 0.001 and 0.001]. The presence of CAKUT as the underlying diagnosis did not affect survival (HR: 1.4; 95% CI: 0.9–2.4; *p* = 0.170) (Table 2). A sensitivity analysis was performed with age included as a continuous variable rather than categorised groups. There was a 0.86-fold decrease in the risk of death for every additional year of age (95% CI: 0.83–0.90).

**Table 2:** Longitudinal changes in serum hemoglobin, albumin, C-reactive protein, and parathyroid hormone levels among patients undergoing peritoneal dialysis and hemodialysis from pre-dialysis to year 5 (CRP: C-reactive protein; ESA: Erythropoietin stimulating agent; IQR: interquartile range, HD: Hemodialysis; PD: Peritoneal dialysis; PTH parathyroid hormone)

Time		Pre-dialysis (*n* = 881)	1 year (*n* = 525)	2 years (*n* = 317)	3 years (*n* = 199)
Serum hemoglobin (g/dL)	PD	8.72 ± 1.66	10.12 ± 1.73	9.94 ± 1.57	10.19 ± 1.62
	HD	10.15 ± 1.90	9.14 ± 1.89	9.02 ± 1.94	8.90 ± 2.11
	*p*	< 0.001	< 0.001	< 0.001	< 0.001
ESA usage rate (%)	PD	133 (47.2)	118 (51.3)	61 (46.6)	39 (48.8)
	HD	186 (61.5)	209 (68.8)	122 (63.2)	82 (66.7)
	*p*	0.036	< 0.001	0.003	0.011
Serum albumin (g/dL)	PD	3.37 ± 0.75	3.43 ± 0.73	3.52 ± 0.57	3.46 ± 0.63
	HD	3.63 ± 0.62	3.91 ± 0.48	3.97 ± 0.49	3.90 ± 0.55
	*p*	< 0.001	< 0.001	< 0.001	< 0.001
C-reactive protein (mg/dL)	PD	2.01 (IQR 3.30)	0.86 (IQR 2.61)	0.86 (IQR 2.80)	0.80 (IQR 2.86)
	HD	2.90 (IQR 7.55)	2.45 (IQR 7.39)	2.00 (IQR 2.65)	3.27 (IQR 8.88)
	*p*	0.018	0.268	0.322	0.127
PTH level (mg/dL)	PD	449.33 (IQR 445.95)	476.49 (IQR 535.85)	397.01 (IQR 501.85)	412.10 (IQR 519.40)
	HD	270.70 (IQR 434.81)	628.72 (IQR 622.70)	568.05 (IQR777.47)	581.20 (IQR 987.42)
	*p*	0.437	0.568	0.010	0.196

### PD versus HD: laboratory outcomes at baseline and during follow-up

At the initiation of kidney replacement therapy, PD patients had lower levels of hemoglobin 8.72 ± 1.66 g/dL vs. 10.15 ± 1.90 g/dL, *p* < 0.001). However, this trend was reversed after starting dialysis. During the three-year follow-up period, patients on PD had higher haemoglobin levels than those on HD (Table 3). The percentage of patients receiving ESA treatment at 1-, 2-, and 3-year intervals of dialysis was significantly higher in the HD group compared to the PD group (68.8% vs. 51.3%, *p* < 0.001; 63.2% vs. 46.6%; p = 0.003 and 66.7% vs. 48.8%, *p* = 0.011, respectively).

**Table 3:** Effect of dialysis modality on fatality, results of univariable and multivariable Cox proportional hazards models

	Univariable				Multivariable			
	Coefficient	Standard error	HR (95% CI)	*p* value	Coefficient	Standard error	HR (95% CI)	*p* value
Dialysis Modality								
Hemodialysis	−0.020	0.161	1.0 (0.7–1.3)	0.901	1.067	0.223	2.9 (1.9–4.5)	< 0.001
Peritoneal dialysis (Ref)								
Age group								
≤ 6 years	0.981	0.208	2.7 (1.8–4.0)	< 0.001	1.982	0.273	7.3 (4.3–12.4)	< 0.001
6.1–12 years	0.604	0.186	1.8 (1.3–2.6)	0.001	0.731	0.224	2.1 (1.3–3.2)	0.001
> 12 years (Ref)								
Period								
2006–2018	0.452	0.165	1.6 (1.1–2.2)	0.006	0.406	0.190	1.5 (1.04–2.2)	0.032
2019–2022 (Ref)								
Presence of CAKUT								
Yes (Ref)								
No	0.233	0.254	1.3 (0.8–2.1)	0.360	0.356	0.259	1.4 (0.9–2.4)	0.170

*HR* Hazard ratio, *CI* Confidence interval

Multivariable Analysis; Event = 158, Censored = 735, Cases with missing values = 83, Censored cases before the earliest event in a stratum = 6, Omnibus test of model coefficients (*p* value) =  < 0.001

In PD patients, serum albumin levels were consistently lower both at the initiation of dialysis and throughout the entire 3-year follow-up period (*p* value was < 0.001 for all years)(Table 3). Median C-reactive protein (CRP) levels were similar over the 3-year follow-up period between the HD and PD groups (Table 3).

There was no significant difference in serum PTH levels between the HD and PD groups at dialysis initiation and during the first year of treatment; however, during the second year, PTH levels were higher in the HD group compared with the PD group (*p* = 0.010) (Table 3).

## Discussion

This nationwide study provides comprehensive survival data for pediatric dialysis patients in Türkiye over a 17-year period. PD was associated with significantly better 3-year survival compared to HD even after adjusting for age, underlying diagnosis, and treatment era. For both study periods (2006–2018 and 2019–2022), HD and PD rates were comparable among pediatric patients > 6 years of age. Among children ≤ 6 years of age, higher survival was observed in the PD group during the 2019–2022 period. Multivariate analysis further confirmed PD, older age, and more recent dialysis initiation as independent predictors of improved survival.

A 2023 study by Ambarsari et al., which included 1,058 paediatric patients with stage 5 CKD, reported that five-year survival was significantly better in those treated with PD than in those treated with HD. The study also found no significant difference in overall survival between the two time periods examined (2000–2010 vs. 2011–2020), suggesting that survival outcomes have remained stable over the past two decades [16]. Consistent with our findings, data from a cohort of 6,473 children with stage 5 CKD indicated that patients first treated with HD had a higher risk of death than PD patients (adjusted HR 1.39; 95% CI 1.06–1.82), and the disparity was greatest in the initial year of treatment (adjusted HR 1.70; 95% CI 1.22–2.38) [6]. However, there are also studies suggesting comparable survival outcomes between PD and HD in the pediatric population. According to the 2022 USRDS data in children and adolescents, the 5-year survival probability was 81% for those on HD and 86% for those on PD; this difference was not statistically significant (*p* = 0.86) [10]. Similarly, the Taiwan Renal Registry reported similar mortality rates for HD and PD patients [14], as summarized in Table 4.

**Table 4:** Summary of kidney replacement therapy survival outcomes in different countries

Ref	Year	Study period	Country/Cohort	N	Patient group	Comparison (PD vs. HD)	Outcome measure
Our study	–	2006–2022	Türkiye	1002	HD, PD	PD is better	25.8 per 1000 patient-years (mortality)
Ambarsari et al. [16]	2023	2000–2020	Australia and New Zealand	1058	HD, PD, KTx	PD is better	1.2 per 100 patient-years (mortality)
Hirano et al. [4]	2023	2006–2013	Japanese nationwide cohort study	701	HD, PD, KTx	NR	5-year survival: 92.5%
Hamilton et al. [12]	2017	1999–2008	UK Renal Registry	3370	HD, PD, KTx	No difference	5-year survival: 91.7%
Chesnaye et al. [6]	2016	2000–2013	European pediatric dialysis patients	6473	HD, PD	PD is better	28.0 per 1000 patient years (mortality)
Mitsnefes et al. [5]	2013	1990–2010	United States Renal Data System	23,401	HD, PD	PD is better	38.6 per 1000 patient-years in children starting dialysis > 5 years (mortality)
Lin et al. [14]	2012	1995–2004	Taiwan Renal Registry	475	HD, PD	No difference	24.66 per 1000 patient-years (mortality)
Groothoff et al. [3]	2002	1972–1992	Dutch cohort studdy	381	HD, PD, KTx	PD is better	1.57 per 100 patient-years (mortality)

*HD* Hemodialysis, *KTx* Kidney transplantation, *PD* Peritoneal dialysis, *NR* Not reported

In this study, which reflects pediatric dialysis data from our country, the mortality rate was 25.8 per 1,000 patient-years. In our country, Bakkaloglu et al.'s study in 2005 showed the overall mortality rate for PD patients was 83 deaths per 1,000 patient-years, while the 5-year survival rate was 70% [15]. Comparative analysis among 7,108 pediatric patients undergoing HD, PD, or kidney transplantation, conducted in 32 countries of Europe, demonstrated a crude 5-year mortality of 15.8 per 1,000 patient-years [9]. That study identified higher mortality estimates for patients from Russia (35.2), Poland (39.9), Romania (47.4), and Bulgaria (68.6) compared with the combined estimate for all Western European countries [9]. In a cohort of 475 pediatric patients from the Taiwan Renal Registry, the mortality rate was 24.66 per 1,000 patient-years [14].

In our cohort, older age was found to be an independent predictor of a significantly lower mortality risk. The Italian Paediatric Chronic Peritoneal Dialysis Registry reported that 84 paediatric patients who started PD before 1 year of age were evaluated [17]. Of these, eight died at a median age of 12.3 months, after a median of 11.8 months on chronic peritoneal dialysis [17]. The United States Renal Data System reported that at 5 years, the survival probability was 0.78 for children under 1 year of age, 0.89 for those aged 1–5 years, and 0.96 for those aged ≥ 6 years [10]. In the single-centre experience reported from Brazil, which included pediatric dialysis patients, the 5-year survival rate was 74.2% (95% CI: 62.2–86.3%) for those aged 6–19 years, and 37.2% (95% CI: 2.6–71.8%) for those aged 0–5 years [18]. According to the data from the Australia and New Zealand Dialysis and Transplant Registry that examined pediatric patients receiving kidney replacement therapy, the 5-year survival probabilities were 73% for children younger than 1 year of age, 79% for children aged 1–4 years, and 87% for those aged 5–9 years [8].

Our study also demonstrated that serum hemoglobin levels were higher and erythropoiesis-stimulating agent (ESA) requirements were lower in PD patients compared to HD patients. A meta-analysis of adult patients with stage 5 CKD found higher mean hemoglobin levels in those on PD (SMD = 0.56, 95% CI: 0.07–1.06; *p* < 0.05) [19]. In a study of 300 children and adolescents on dialysis, the first-year hemoglobin level was significantly higher in PD patients compared with HD patients (115.2 ± 18.5 g/dL vs. 107.5 ± 17.5 g/dL, *p* = 0.01) [20]. A multicenter study by Bakkaloğlu et al. from our country showed that HD patients were more often anemic (53.3% vs. 35%, *p* = 0.01) and it was harder for them to achieve the target hemoglobin level (< 10 g/dL) [21].

This study has several limitations. Firstly, it is retrospective in nature. Consequently, other potential risk factors that could influence mortality, such as fluid status, nutritional characteristics and infection episodes, could not be evaluated. Secondly, information on kidney transplant patients was unavailable, since transplant data are not included in the national registry used for this study. Consequently, it was not possible to determine the number of patients who underwent transplantation during the study period, which may limit the interpretation of long-term outcomes. In addition, the follow-up period was relatively short, with approximately half of the patients having data available for only two years. This limited follow-up period may have affected the assessment of long-term survival. Furthermore, the aetiology of primary kidney disease was unknown in nearly half of the patients due to the absence of a standardised primary renal disease (PRD) coding system. Finally, the comparison periods (2006–2018 and 2019–2022) were not of equal length, which may have influenced the distribution of patients and created an imbalance between the groups. Although it has some limitations, we consider it of value since it represents nationwide data with a large set of laboratory parameters during the follow-up and will contribute to the literature.

In conclusion, in this nationwide cohort of paediatric dialysis patients, PD was associated with significantly better three-year survival rates than HD, particularly among younger children and in the most recent treatment era. These findings support the preferential use of PD in selected paediatric populations to improve long-term outcomes. To validate and expand upon these findings, future prospective and randomized studies are warranted.

## Supplementary Information

Below is the link to the electronic supplementary material.

ESM 1 Graphical abstract (PPTX 260 KB)

## Data Availability

The data that support the fndings of this case are available from the corresponding author (G.K.A.).
