# Supplementary material for: Survival of paediatric dialysis patients in Türkiye over a three-year follow-up: findings from national database
Source: Pediatr Nephrol. 2026 Apr 8;41(9):2989–99. doi: 10.1007/s00467-026-07264-z (PMC13424471; doi:10.1007/s00467-026-07264-z)
Supplement: Supplementary file 1 — Graphical abstract (PPTX 260 KB) [file 467_2026_7264_MOESM1_ESM.pptx]

## Slide 1
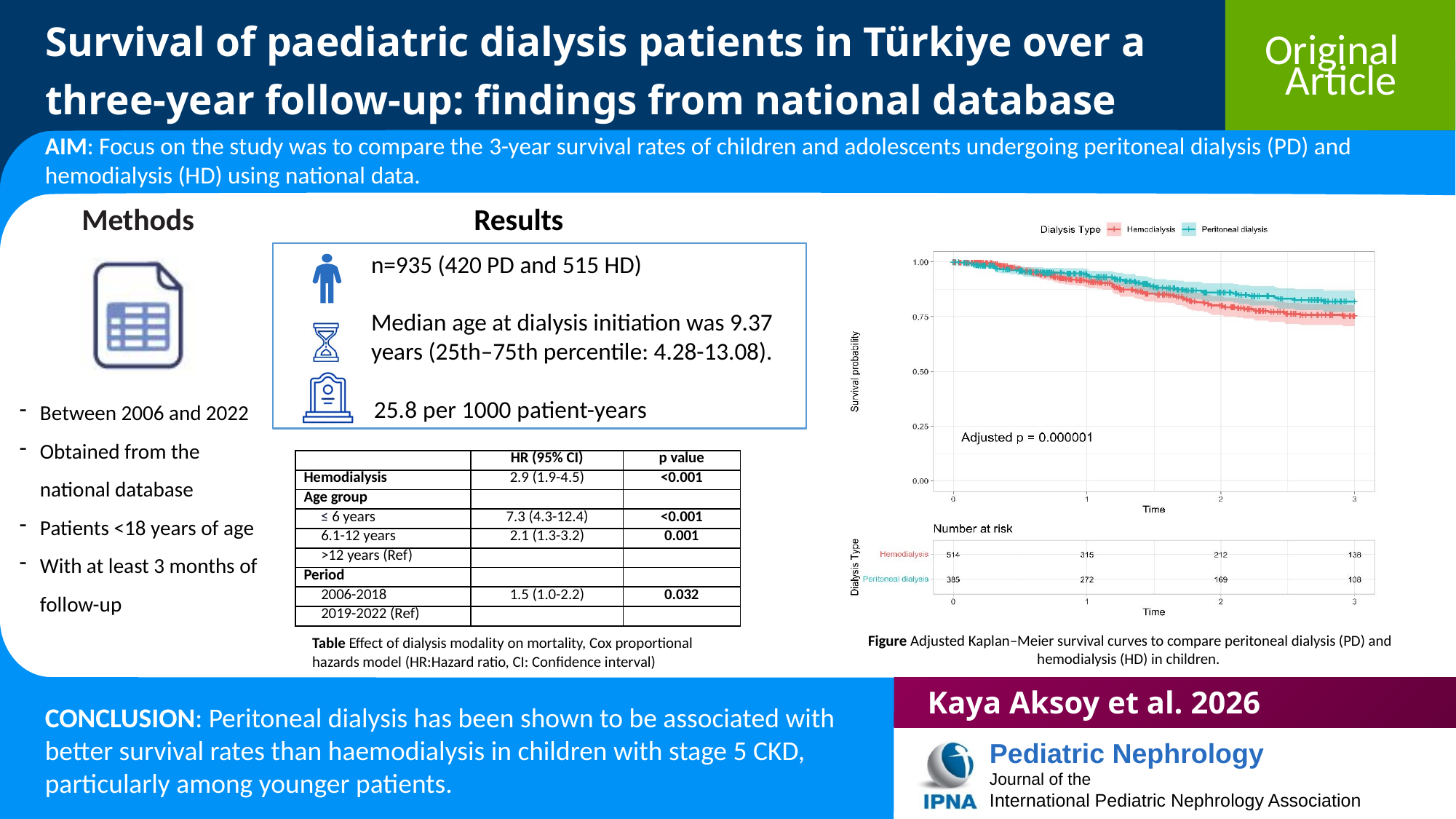

Survival of paediatric dialysis patients in Türkiye over a three-year follow-up: findings from national database
AIM: Focus on the study was to compare the 3-year survival rates of children and adolescents undergoing peritoneal dialysis (PD) and hemodialysis (HD) using national data.
Methods
Results
 n=935 (420 PD and 515 HD)
 Median age at dialysis initiation was 9.37
 years (25th–75th percentile: 4.28-13.08).
 25.8 per 1000 patient-years
Between 2006 and 2022
Obtained from the national database
Patients <18 years of age
With at least 3 months of follow-up
| | HR (95% CI) | p value |
| --- | --- | --- |
| Hemodialysis | 2.9 (1.9-4.5) | <0.001 |
| Age group | | |
| ≤ 6 years | 7.3 (4.3-12.4) | <0.001 |
| 6.1-12 years | 2.1 (1.3-3.2) | 0.001 |
| >12 years (Ref) | | |
| Period | | |
| 2006-2018 | 1.5 (1.0-2.2) | 0.032 |
| 2019-2022 (Ref) | | |
Figure Adjusted Kaplan–Meier survival curves to compare peritoneal dialysis (PD) and hemodialysis (HD) in children.
Table Effect of dialysis modality on mortality, Cox proportional hazards model (HR:Hazard ratio, CI: Confidence interval)
Kaya Aksoy et al. 2026
CONCLUSION: Peritoneal dialysis has been shown to be associated with better survival rates than haemodialysis in children with stage 5 CKD, particularly among younger patients.
